# Compassionate Use of Ripretinib for Patients With Metastatic Gastrointestinal Stromal Tumors: Taiwan and Hong Kong Experience

**DOI:** 10.3389/fonc.2022.883399

**Published:** 2022-06-29

**Authors:** Li-Ching Lin, Wen-Kuan Huang, Chueh-Chuan Yen, Ching-Yao Yang, Meng-Ta Sung, Natalie S. M. Wong, Daniel T. T. Chua, Sarah W. M. Lee, Jen-Shi Chen, Chun-Nan Yeh

**Affiliations:** ^1^ Department of Surgery and GIST Team, Chang Gung Memorial Hospital at Linkou, Chang Gung University College of Medicine, Taoyuan, Taiwan; ^2^ Division of Hematology-Oncology, Department of Internal Medicine and GIST Team, Chang Gung Memorial Hospital at Linkou, Chang Gung University College of Medicine, Taoyuan, Taiwan; ^3^ Division of Clinical Research, Department of Medical Research and Division of Medical Oncology, Center for Immuno-oncology, Department of Oncology, Taipei Veterans General Hospital, Taipei, Taiwan; ^4^ National Yang Ming Chiao Tung University School of Medicine, Taipei, Taiwan; ^5^ Department of Surgery, National Taiwan University Hospital and College of Medicine, National Taiwan University, Taipei, Taiwan; ^6^ Department of Oncology, Mennonite Christian Hospital, Haulien, Taiwan; ^7^ Department of Clinical Oncology, Tuen Mun Hospital, Hong Kong, Hong Kong SAR, China; ^8^ Department of Medicine, Hong Kong Sanatorium and Hospital, Hong Kong, Hong Kong SAR, China; ^9^ Department of Clinical Oncology, Pamela Youde Nethersole Eastern Hospital, Hong Kong, Hong Kong SAR, China

**Keywords:** ripretinib, GIST, compassionate use, advanced, metastatic

## Abstract

**Background:**

Ripretinib was recently approved for the fourth-line targeted therapy for advanced gastrointestinal stromal tumor (GIST) refractory to imatinib, sunitinib, and regorafenib based on the pivotal INVICTUS phase III study. The INVICTUS study demonstrated significantly improved median progression-free survival (PFS) of 6.3 months and an overall survival (OS) insignificant benefit of ripretinib of 15.1 months as compared with placebo in 85 patients with advanced metastatic GIST. However, treatment outcome for the Chinese population, including in Taiwan and Hong Kong, was lacking.

**Material and Method:**

A compassionate study regarding ripretinib use for patients with advanced/metastatic GIST was conducted from March 2020 to March 2021 to assess the treatment efficacy and safety in Taiwan and Hong Kong patients.

**Result:**

Twenty evaluable patients (16 men and 4 women) with heavily pretreated metastatic GIST receiving ripretinib from March 2020 to March 2021 were enrolled to evaluate the treatment outcome. The response and clinical benefit rates to ripretinib were 25% (5/20) and 60% (12/20), respectively. The median PFS and OS in this compassionate cohort receiving ripretinib were 6.1 months and not reachable, respectively. Albumin less than 3.5 and disease progression after ripretinib use were the two independent unfavorable factors for PFS. There were 14 out of 20 (70%) experiencing any grade adverse event (AE). Loss of hair is the most common grade I to II AE with an incidence of 55%. Grade III AEs included diarrhea, skin rash, and anemia with one patient (5%) for each AE.

**Conclusions:**

Late-line ripretinib use in pretreated Taiwan and Hong Kong patients with advanced GIST showed efficacy consistent with the INVICTUS study. Albumin less than 3.5 and disease progression after ripretinib use were the two independent unfavorable factors for PFS. Ripretinib is generally tolerable, with loss of hair being the most common AE.

## Introduction

Gastrointestinal stromal tumors (GISTs) are the most common mesenchymal tumors of the gastrointestinal tract. GIST is addicted to mutations in the oncogenes KIT or PDGFRA in a mutually exclusive manner, which demonstrated a model for targeted drug development (1). *KIT/PDGFRA* wild-type GISTs are deficient in succinate dehydrogenase or have alterations in the mitogen-activated protein kinase signaling pathway (2, 3). The approval of second-line sunitinib and third-line regorafenib has substantially changed the management of advanced GISTs, leading to the standard sequences of tyrosine kinase inhibitors (TKIs) after imatinib failure (4, 5). First-line imatinib is effective and has a favorable safety profile, whereas second-line sunitinib and third-line regorafenib are reportedly less effective. The median progression-free survival (PFS) of patients receiving sunitinib was approximately 6–9 months (6, 7). Studies have indicated that third-line regorafenib exerted a weaker antitumor effect, resulting in a median PFS of 4.4–4.8 months (5, 8). The management of GISTs in the late-line setting in terms of overcoming the resistance of TKIs was an unmet need until the recent approval of ripretinib.

Ripretinib is a novel, type II tyrosine switch control inhibitor targeting a broad spectrum of primary and TKI-resistant *KIT* and *PDGFRA* variants (9). Blockage of both switches (kinase switch pocket and activation loop) induces kinases to stabilize in the inactive state and prevents them from adopting an active conformation. A phase I study reported promising results of ripretinib when used as the second-, third-, and fourth-line drug, with median PFS ranging from 5.5 to 10.7 months. The phase III INVICTUS study explored and compared the efficacy and safety of ripretinib (150 mg once daily) with those of placebo in advanced GIST refractory to at least three-line TKIs. Ripretinib demonstrated higher efficacy when used as the fourth-line drug with an overall response and median PFS of 9% and 6.3 months, respectively (10). The safety profile of ripretinib is generally acceptable, and the most common adverse events (AEs) include alopecia, fatigue, and nausea. On the basis of the aforementioned results, ripretinib was approved for use as the fourth-line drug by the US Food and Drug Administration (FDA) for patients with advanced GIST after treatment with three or more TKIs, including imatinib, sunitinib, and regorafenib.

In the INVICTUS study, races were classified as white and non-white. The treatment outcomes of the patients of Chinese ethnicity, including Taiwanese and Hong Kongers, were not examined. Therefore, we conducted this multi-institutional study to investigate the antitumor efficacy and safety of ripretinib. In addition, we performed a literature review to compare the treatment outcomes of ripretinib observed in our study with those reported in previous studies.

## Methods

### Patients, Study Design, and Efficacy Evaluation

From March 2020 to March 2021, a total of 22 patients who received a histological diagnosis of advanced GIST were administered ripretinib therapy on a compassionate-use basis in five hospitals (one from Hong Kong and four from Taiwan). We collected clinical data prospectively and reviewed this cohort retrospectively. Although ripretinib was approved by the US FDA in May 2020, it is not yet covered by Taiwan’s National Health Insurance program. Thus, the use of ripretinib on a compassionate-use basis was started. The patients were administered a dose of ripretinib of 150 mg orally per day, and this dose was continued until unmanageable toxicity or disease progression. At the physician’s discretion, ripretinib was continued despite the radiographic evidence of disease progression as long as the clinical benefit was observed. By contrast, dose reduction or interruption was considered in the presence of drug-associated AEs. Dose reescalation was allowed after the resolution of AEs. Performance status, weight, complete blood count, and serum chemistry including hepatic and renal functions were regularly checked during each visit. Complete response (CR), partial response (PR), stable disease (SD), and disease progression were evaluated using the Response Evaluation Criteria in Solid Tumors (RECIST), version 1.1 (11). The clinical benefit rate is defined as the percentage of patients who have achieved CR and PR. CT scans were performed every 3 months to evaluate the response. Time to response (TTR = the time interval from the administration of ripretinib to the observation of the most favorable response) is defined as the time interval from the administration of a drug to the observation of the most favorable response during the treatment course. Time to progression (TTP = the time interval from ripretinib administration to disease progression) is defined as the time interval from the administration of a drug to the evidence of disease progression during the treatment course. PFS was calculated as the period from the initiation of ripretinib treatment to disease progression or death. Overall survival (OS) was calculated as the period from ripretinib administration to death or September 2021 (the end of follow-up in this study). AEs of ripretinib were evaluated in accordance with the National Cancer Institute Common Terminology Criteria for Adverse Events (version 4.0). The study protocol was approved by the Institutional Review Board of Chang Gung Memorial Hospital (IRB number: 201601745B0, Taoyuan, Taiwan).

### Statistical Analysis

All descriptive statistics are presented as the percentage or the mean ± standard deviation. All numerical data were compared using an independent two-sample t-test. Pearson’s χ^2^ and Fisher’s exact tests were used to analyze nominal variables. Survival analysis was performed using the Kaplan–Meier method with the log-rank test. The significance of several variables for survival was analyzed by conducting univariate and multivariate Cox regression analyses. The variables were age (<60 vs. ≥60 years), sex, Eastern Cooperative Oncology Group (ECOG) performance score, tumor response, primary site, and metastatic site of GIST, and laboratory data [white blood cell count, neutrophil-to-lymphocyte ratio, platelet count, platelet-to-lymphocyte ratio (PLR), and hemoglobin and albumin levels]. The variables that were determined to be statistically significant in univariate analysis were further analyzed using a Cox multivariate proportional hazards model. We used the enter selection method to select the most relevant prognostic factors. Factors that remained significant were included in the final model. All statistical analyses were performed using SPSS version 20.0 (IBM Corp). A *p*-value of <0.05 was considered statistically significant.

## Results

### Clinical Features

Two patients were excluded from survival analysis: one patient developed disease progression within 1 month of ripretinib use, and the other patient was lost to follow-up. A total of 20 patients who exhibited disease progression or intolerance to prior treatment with imatinib, sunitinib, and regorafenib were included in the final analysis. Of the 20 patients, 16 were men and 4 were women, and 14 were from Taiwan and 6 were from Hong Kong. These 20 patients had an ECOG performance score of ≤3 with sufficient hepatic, renal, and hematological functions. The median age of the patients was 61 (range, 33–76) years, and they had advanced inoperable or metastatic GISTs that were treated with ripretinib (**Table 1**). The small bowel was the most common site of GISTs treated with ripretinib (10/20; 50%), and the liver was the leading metastatic site (18/20; 90%), followed by the peritoneum (13/20; 65%).

**Table 1:** Clinicopathological characteristics of patients with advanced GIST treated with ripretinib.

Characteristics	No. of cases	Percentage (%) or median (range)
Gender		
Male	16	80.0
Female	4	20.0
Age (years)	20	61 (33–76)
ECOG		
0	3	15.0
1	13	65.0
2	4	20.0
3	0	0
Body composition		
Weight (kg)	20	64.4 (45.0–91.1)
Height (cm)	20	166.9 (153.0–191.2)
BMI (kg/m^2^)	20	23.4 (16.7–29.8)
BMI		
<18	2	10.0
18–27	15	75.0
>27	3	15.0
Primary site		
Stomach	8	40.0
Small bowel	10	50.0
Rectum	1	5.0
Others	1	5.0
Metastatic site		
Liver	18	90.0
Peritoneum	13	65.0
Others	9	45.0
Median age at the time of		
Diagnosis of GIST	20	51.5 (29–71)
Start of ripretinib	20	64 (33–76)

ECOG, Eastern Cooperative Oncology Group; BMI, body mass index; GIST, gastrointestinal stromal tumor.

### Treatment and Outcomes

Ripretinib (150 mg per day) was administered to all the 20 patients with pretreated metastatic GISTs. **Table 2** presents the findings of the most favorable antitumor response of ripretinib in all the patients with pretreated metastatic GISTs. Overall, five (25%), seven (35%), and eight (40%) patients exhibited PR, SD, and disease progression, respectively (**Table 2**). The clinical benefit rate was 60%. The median TTR for the five patients with PR and seven patients with SD was 2.5 and 2.8 months, respectively. The median TTP was 2.6 months for the 10 patients with disease progression (**Table 3**).

**Table 2:** Treatment outcomes of patients with advanced GIST (n=20).

Characteristics	No. of cases	Percentage (%) or median (range)
Median duration of prior TKI and ripretinib therapy (months)		
Imatinib therapy	20	22.9 (4.2–130.1)
Sunitinib therapy	19	12.0 (1.6–53.1)
Regorafenib therapy	17	5.4 (0.5–49.5)
Ripretinib therapy	20	10.4 (1.4–18.6)
Treatment and outcomes		
Median duration of ripretinib therapy (months)	20	10.4 (1.4–18.6)
Median time to response (months)	20	2.7 (1.4–6.7)
Best response		
PR	5	25.0
SD	7	35.0
PD	8	40.0
Survival status		
Alive	15	75.0
Expire	5	25.0
Last follow-up date	20	2021/9/30
Median follow-up time (months)	20	10.4 (1.4–18.6)

PR, partial response; SD, stable disease; PD, progressive disease; TKI, tyrosine kinase inhibitors.

**Table 3:** Treatment outcome of patients with advanced GIST treated with ripretinib.

Best response	N (%)	Gendermale/female, n	Median ripretinib duration, months	Median TTR/TTP, months
PR	5 (25.0)	4/1	13.2	2.5/8.4
SD	7 (35.0)	4/3	9.3	2.8/6.3
PD	8 (40.0)	8/0	5.2	NA/2.6

PR, partial response; SD, stable disease; PD, progressive disease; TTR, time to response; TTP, time to progression; NA, not available; GIST, gastrointestinal stromal tumor.

### Survival Analysis of Patients With Pretreated Metastatic Gastrointestinal Stromal Tumors Receiving Ripretinib

**Figure 1:**
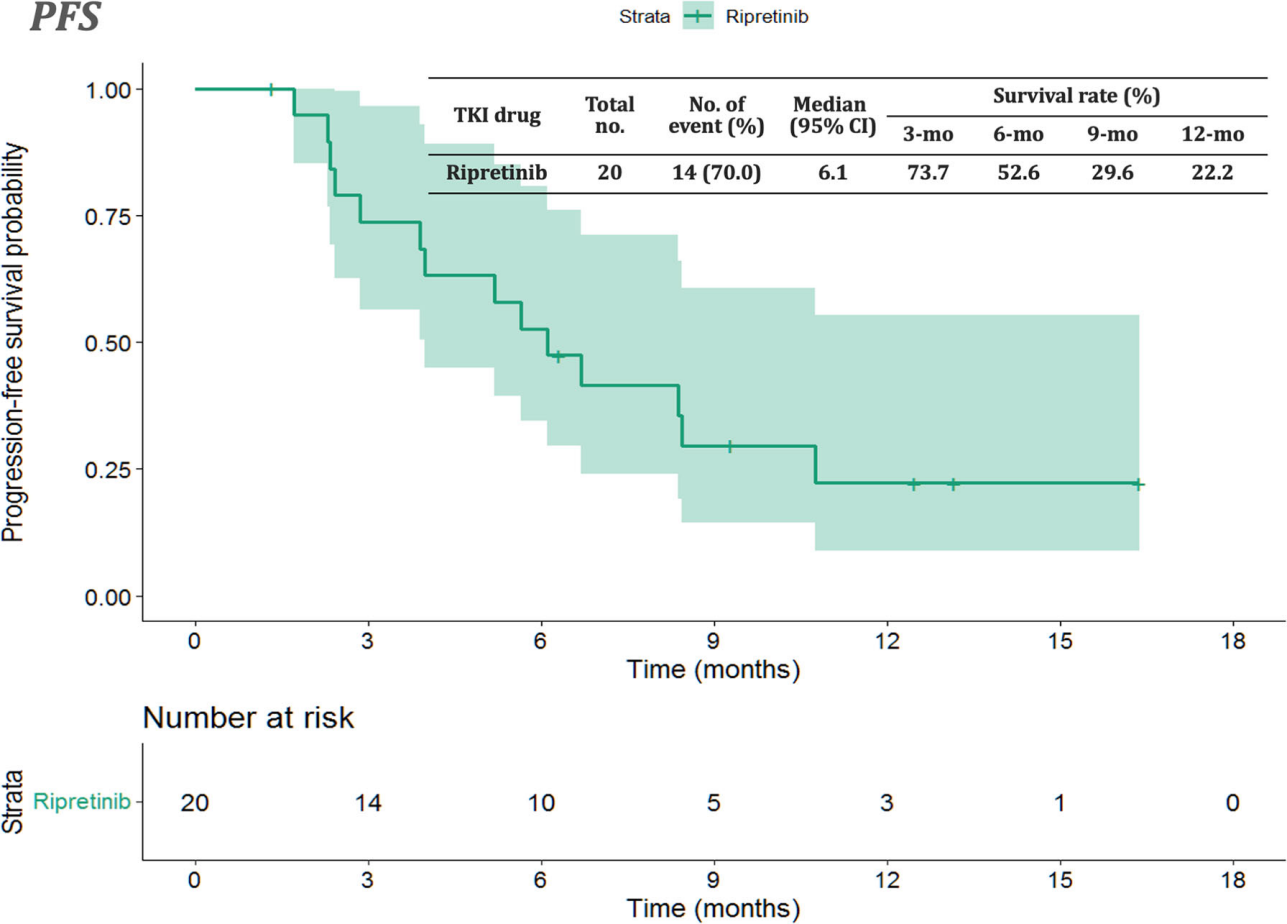
Kaplan–Meier plot of progression-free survival in patients receiving ripretinib. CI: confidence interval.

**Figure 2:**
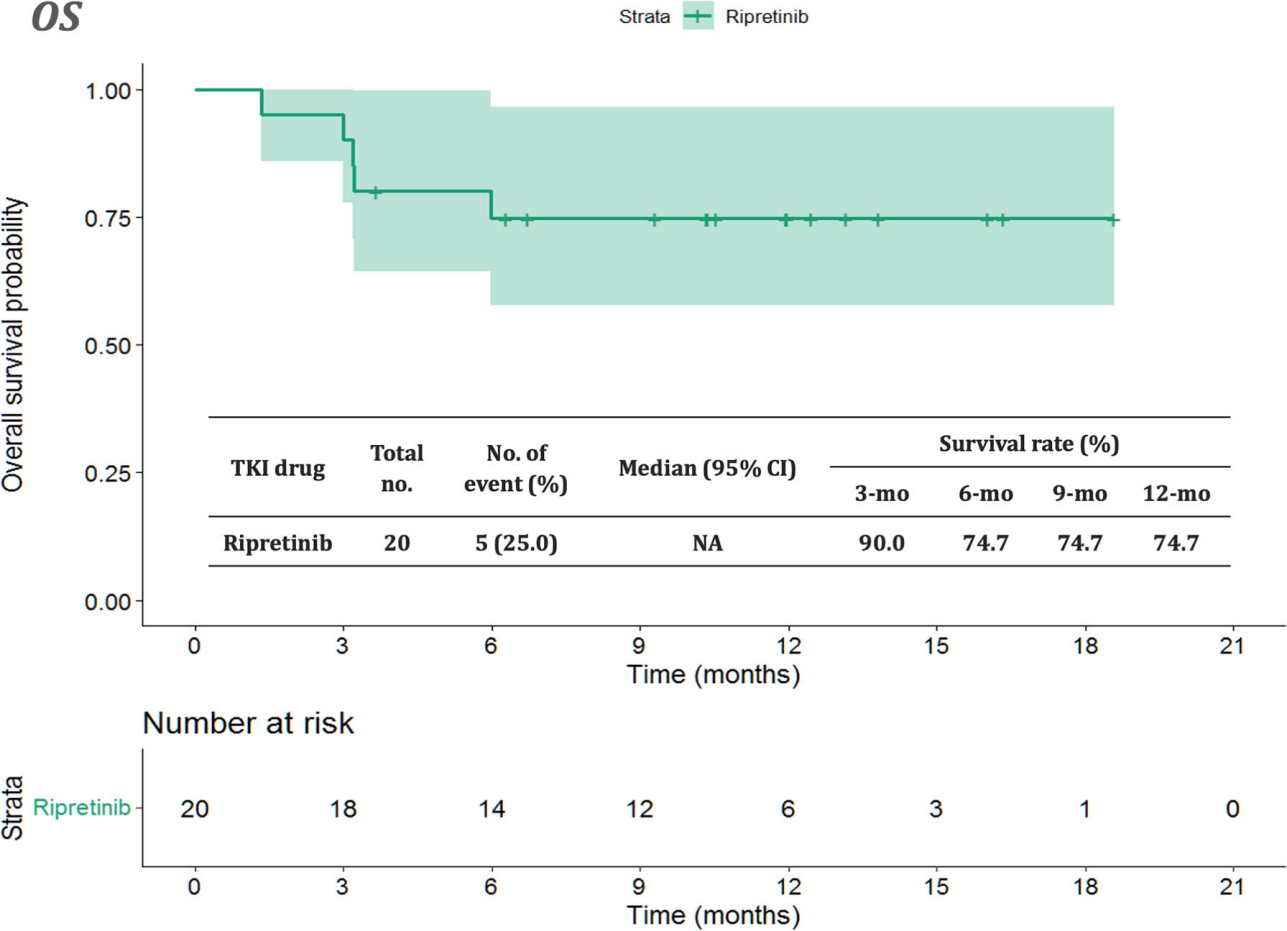
Kaplan–Meier plot of overall survival in patients receiving ripretinib. CI: confidence interval.

**Figure 3:**
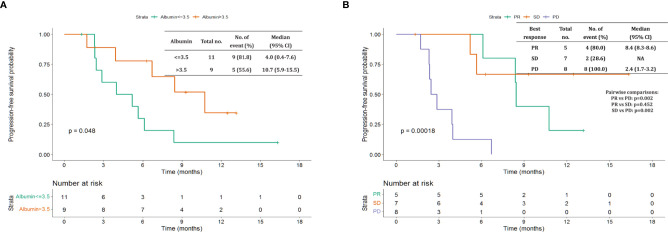
Kaplan–Meier plot of progression-free survival based on pretreatment albumin levels and disease control status. **(A)** Pretreatment albumin level >3.5 vs. ≤3.5 g/dL **(B)** partial response (PR) vs. stable disease (SD) vs. disease progression.

The median follow-up period after ripretinib treatment was 10.4 (range, 1.4–18.6) months. Among the 20 patients, 14 (70%) experienced disease progression during follow-up. The median PFS and OS of the 20 patients were 6.1 months and not reached, respectively (**Figures 1**, **2**). **Tables 4**, **5** summarize findings on the prognostic factors for PFS and OS. The results of univariate survival analysis revealed that female sex, albumin <3.5 g/dl, higher PLR, and disease control after ripretinib use were associated with prolonged PFS after ripretinib treatment (**Table 4**). Serum albumin <3.5 g/dl and the absence of toxicity were unfavorable factors associated with OS in the univariate analysis; however, no significant variable was noted in the multivariate analysis for OS (**Table 5**). The results of the multivariate Cox proportional hazards model revealed that adequate nutritional status with albumin ≥3.5 g/dl and disease control after ripretinib use were the only two independent favorable factors associated with the PFS of the patients with advanced GISTs receiving ripretinib treatment (**Table 4** and **Figures 3A, B**).

**Table 4:** Univariate and multivariate Cox regression analyses in progression-free survival.

Variables	Total no.	Univariate analysis				Multivariate analysis		
		No. of event (%)	Median (months)	95% CI	*p-*Value	Hazard ratio	95% CI	*p-*Value
Gender					0.033			
Male	16	13 (81.2)	10.7			1.10	0.09–13.03	0.941
Female	4	1 (25.0)	5.2	3.0–7.4		Reference		
ECOG PS					0.100			
0/1	16	11 (68.8)	6.7	1.9–11.5		Reference		
2	4	2 (75.0)	4.0	1.3–6.7		0.55	0.07–4.14	0.563
Albumin (g/dl)					0.048			
≤3.5	11	9 (81.8)	4.0	0.4–7.6		6.24	1.12–34.70	0.036
>3.5	9	5 (55.6)	10.7	5.9–15.5		Reference		
Lymphocyte					0.055			
≤2,432	18	12 (66.7)	6.7	3.3–10.1		Reference		
>2,432	2	2 (100.0)	2.4	–		0.23	0.01–7.96	0.414
PLR					0.003			
≤86.44	3	3 (100.0)	2.4	2.2–2.6		10.63	0.40–279.89	0.157
>86.44	17	11 (64.7)	6.7	2.7–10.7		Reference		
Best response					<0.001			
PR	5	4 (80.0)	8.4	8.3–8.6		Reference		
SD	7	2 (28.6)	NA			0.30	0.05–1.99	0.211
PD	8	8 (100.0)	2.4	1.7–3.2		11.62	1.83–73.67	0.009

Univariate variables with p-values ≤0.1 were included in the multivariate Cox regression model.

PS, performance status; PLR, platelet-to-lymphocyte ratio; PR, partial response; SD, stable disease; PD, progressive disease; ECOG, Eastern Cooperative Oncology Group.; NA, not available.

**Table 5:** Univariate and multivariate Cox regression analyses of prognostic factors in overall survival.

Variables	Total no.	Progression-free survival				Multivariate analysis*		
		No. of event (%)	Median (months)	95% CI	*p-*Value	Hazard ratio	95% CI	*p-*Value
Albumin (g/dl)					0.022			
≤3.5	11	5 (45.5)	NA			5.53	0.29–815.92	0.255
>3.5	9	0	NA			Reference		
PLR					0.074			
≤86.44	3	2 (66.7)	3.2	3.2–3.3		5.45	0.79–40.29	0.083
>86.44	17	3 (17.6)	NA			Reference		
Any toxicity					0.003			
No	6	4 (66.7)	3.2	0.1–6.8		3.90	0.68–41.18	0.131
Yes	14	1 (7.1)	NA			Reference		

Univariate variables with p-values ≤ 0.1 were included in the multivariate Cox regression model.

PLR, platelet-to-lymphocyte ratio.

^*^Cox regression using Firth’s correction: a solution to the problem of monotone likelihood in Cox regression.NA, not available.

### Literature Review for Comparing the Effect of Ripretinib on Gastrointestinal Stromal Tumor Globally

We conducted a global literature review of patients with GIST who received ripretinib treatment to compare the findings of the present study cohort with those reported in previous studies. The clinical efficacy of ripretinib observed in our study is similar to that reported in the INVICTUS trial including a few Asian patients (six patients from Singapore and three patients from Australia; **Table 6**).

**Table 6:** Literature review concerning the treatment outcomes of patients with metastatic gastrointestinal stromal tumor treated with ripretinib.

No.	Author, year	Study design	Published	Area	No. of patients	PFS, months	OS, months	CBR, %	Grade 3 adverse events	Prognostic factors for PFS	Prognostic factors for OS
1	Jean-Yves Blay, 2020 (10)	Randomized controlled trial	*Lancet Oncology*	Global [America, Europe, Asia (Singapore and Australia)]	129	6.3	15.1	51	Lipase increase, HTN, fatigue, hypophosphatemia	N/A	N/A
2	Chao Wu, 2021	Case report	*Annals of Palliative Medicine*	China	1	7	7	0	Nil	N/A	N/A
3	The present study	Case-series study		Taiwan and Hong Kong	20	6.1	NA	60	Anemia, diarrhea, and dermatological toxicity	Albumin and disease control	NA

PFS, progression-free survival; OS, overall survival; CBR, clinical benefit rate; HTN, hypertension; NA, not available.

### Safety

Safety was evaluated in all 20 patients. **Table 7** summarizes the hematological and non-hematological AEs in the patients. Of the 20 patients, 14 (70%) experienced AE of any grade. Alopecia was the most common grade I to II AE, with the highest incidence being 55%. Diarrhea, skin rash, and anemia were the most common grade III AEs with the incidence of each AE being 5.0%. No grade IV/V events were noted during the follow-up period.

**Table 7:** Adverse events.

Adverse events	Any grade (n) (%)	Grade I–II (n) (%)	Grade III (n) (%)
Any toxicity	14 (70.0)	11 (55.0)	3 (15.0)
Alopecia	11 (55.0)	11 (55.0)	0
Anemia	6 (30.0)	5 (25.0)	1 (5.0)
Other dermatologic toxicity	6 (30.0)	5 (25.0)	1 (5.0)
Constipation	5 (25.0)	5 (25.0)	0
Hand–foot syndrome/PPES	5 (25.0)	5 (25.0)	0
Myalgia	5 (25.0)	5 (25.0)	0
Diarrhea	4 (20.0)	3 (15.0)	1 (5.0)
Fatigue	3 (15.0)	3 (15.0)	0
Anorexia	2 (10.0)	2 (10.0)	0
HTN	2 (10.0)	2 (10.0)	0
Hyperbilirubinemia	2 (10.0)	2 (10.0)	0
Muscle spasm	2 (10.0)	2 (10.0)	0
Headache	1 (5.0)	1 (5.0)	0
Hypophosphatemia	1 (5.0)	1 (5.0)	0
Nausea/vomiting	1 (5.0)	1 (5.0)	0
Sore throat	1 (5.0)	1 (5.0)	0
Syncope	1 (5.0)	1 (5.0)	0
Thrombocytopenia	1 (5.0)	1 (5.0)	0
Upper gastrointestinal bleeding	1 (5.0)	1 (5.0)	0
Abdominal pain	0	0	0
Edema	0	0	0

PPES, palmar-plantar erythrodysesthesia; HTN, hypertension.

## Discussion

This multi-institutional study investigated the outcomes of ripretinib treatment administered on a compassionate-use basis in patients from Taiwan and Hong Kong with heavily pretreated metastatic GISTs. Our study revealed several points of interest regarding ripretinib use in advanced GISTs.

First, the median PFS and OS of the 20 patients were 6.1 and not reached, respectively. The clinical efficacy of ripretinib observed in our study is similar to that reported in the INVICTUS trial (10). The treatment efficacy in our study is even more favorable because of a median time lag of 2.7 months (i.e., treatment-free period) between the application of ripretinib on a compassionate-use basis and the first dose of ripretinib. Although tissue re-biopsy is usually impractical for patients with heavily pretreated metastatic GISTs, molecular profiling can be conducted to identify *KIT/PDGFRA* variants that respond to ripretinib. The findings of molecular profiling can be valuable for drug development.

Second, in terms of ripretinib-associated AEs, the toxicity profiles of all the patients in our study are similar to those of the patients in the INVICTUS trial (10). Alopecia was the most frequently observed AE in the present study. Although alopecia is not a severe AE, this condition is associated with substantial unfavorable consequences, such as psychological and physical impact; therefore, dose reduction may be suggested (12). Of note, alopecia upon targeted drug use is relatively rare, suggesting this unique feature of ripretinib use. The calculated overall incidence of all-grade alopecia was 14.7% (95% CI: 12.6%–17.2%), which is lower than that of bortezomib (2.2%; 95% CI: 0.4%–10.9%) and higher than that of vismodegib (56.9%; 95% CI: 50.5%–63.1%). An increased risk of all-grade alopecia [relative risk (RR): 7.9; 95% CI: 6.2–10.09, p ≤ 0.01] compared with placebo was observed; however, the risk was lower when compared with chemotherapy [(RR: 0.32; 95% CI: 0.2–0.55, p ≤ 0.01)] (12). Thus, ripretinib is regarded as the common molecular targeted agent that usually causes alopecia.

In the present study, two independent prognostic factors for PFS were identified: pretreatment albumin level and disease control by ripretinib. Disease control by ripretinib was consistently observed to be an independent factor for prolonged PFS in the present study; this finding agrees with those of previous studies in which disease control status was consistently demonstrated to be a favorable factor for PFS and OS (8, 13).

A previous study reported that higher pretreatment serum albumin levels were not a favorable factor associated with prolonged OS after the treatment of patients with advanced GISTs with two lines of TKIs (8). In a meta-analysis examining studies on the cancer of the gastrointestinal tract, 26 of 29 studies reported that higher serum albumin levels were associated with prolonged survival (14). Future basic or translational studies should be conducted to elucidate molecular mechanisms underlying the association between serum albumin levels and survival.

For patients who demonstrated disease progression following treatment with 150 mg of ripretinib, an additional clinical benefit may be obtained by increasing the dose of ripretinib to 300 mg (15). However, the standard treatment beyond ripretinib failure is uncertain; therefore, referral to a clinical trial is strongly recommended for patients still experiencing disease progression after an increase in ripretinib dose.

This study has some limitations. First, this study is observational in nature, which may lead to underreported AEs. Second, because of the short follow-up period, the median OS could not be determined. Finally, because we did not perform the profiling of genetic variants, potential response biomarkers could not be identified.

## Conclusions

In this real-world study, the efficacy and safety profile of the late-line use of ripretinib in patients with advanced GIST from Taiwan and Hong Kong are comparable with those reported in the INVICTUS trial. Higher pretreatment albumin levels and satisfactory disease control predicted favorable PFS.

## Data Availability Statement

The original contributions presented in the study are included in the article/**Supplementary Material**. Further inquiries can be directed to the corresponding authors.

## Ethics Statement

The studies involving human participants were reviewed and approved by The Institutional Review Board of the Chang Gung Memorial Hospital (IRB number: 201601745B0, Taoyuan, Taiwan). The patients/participants provided their written informed consent to participate in this study.

## Author Contributions

C-NY designed the study. L-CL and C-NY collected the data and reviewed the literature. L-CL, W-KH, and C-NY wrote the manuscript. J-SC, C-CY, C-YY, M-TS, SW, and DC provided the patient data, interpreted the data, and critically revised the manuscript for important intellectual content. All authors read and approved the final manuscript.

## Funding

This work was supported in part by the Chang Gung Memorial Hospital CMRPG3J0971~3 CORPG3J0251~3. We thank to Zai Lab (Taiwan) Limited for the educational grant to Taiwan Society of Molecular Medicine. The funder Zai Lab (Taiwan) was not involved in the study design, collection, analysis, interpretation of data, the writing of this article, or the decision to submit it for publication.

## Conflict of Interest

The authors declare that the research was conducted in the absence of any commercial or financial relationships that could be construed as a potential conflict of interest.

## Publisher’s Note

All claims expressed in this article are solely those of the authors and do not necessarily represent those of their affiliated organizations, or those of the publisher, the editors and the reviewers. Any product that may be evaluated in this article, or claim that may be made by its manufacturer, is not guaranteed or endorsed by the publisher.

## Supplementary Material

Supplementary Table 1 Univariate analysis of prognostic factors in progression-free survival and overall survival.
