# Corrigendum: Compassionate use of ripretinib for patients with metastatic gastrointestinal stromal tumors: Taiwan and Hong Kong experience

**DOI:** 10.3389/fonc.2022.1028118

**Published:** 2022-10-13

**Authors:** Li-Ching Lin, Wen-Kuan Huang, Chueh-Chuan Yen, Ching-Yao Yang, Meng-Ta Sung, Natalie S.M. Wong, Daniel T. T. Chua, Sarah W. M. Lee, Jen-Shi Chen, Chun-Nan Yeh

**Affiliations:** ^1^ Department of Surgery and GIST Team, Chang Gung Memorial Hospital at Linkou, Chang Gung University College of Medicine, Taoyuan, Taiwan; ^2^ Division of Hematology-Oncology, Department of Internal Medicine and GIST Team, Chang Gung Memorial Hospital at Linkou, Chang Gung University College of Medicine, Taoyuan, Taiwan; ^3^ Division of Clinical Research, Department of Medical Research and Division of Medical Oncology, Center for Immuno-oncology, Department of Oncology, Taipei Veterans General Hospital, Taipei, Taiwan; ^4^ National Yang Ming Chiao Tung University School of Medicine, Taipei, Taiwan; ^5^ Department of Surgery, National Taiwan University Hospital and College of Medicine, National Taiwan University, Taipei, Taiwan; ^6^ Department of Oncology, Mennonite Christian Hospital, Haulien, Taiwan; ^7^ Department of Clinical Oncology, Tuen Mun Hospital, Hong Kong, Hong Kong SAR, China; ^8^ Department of Medicine, Hong Kong Sanatorium and Hospital, Hong Kong, Hong Kong SAR, China; ^9^ Department of Clinical Oncology, Pamela Youde Nethersole Eastern Hospital, Hong Kong, Hong Kong SAR, China

**Keywords:** ripretinib, GIST, compassionate use, advanced, metastatic

In the published article, there was an error in affiliations 3 and 4. Instead of “3. Division of Clinical Research, Department of Medical Research and Division of Medical Oncology, Center for Immuno-oncology, Taipei, Taiwan, 4 Department of Oncology, Taipei Veterans General Hospital and National Yang Ming Chiao Tung University School of Medicine, Taipei, Taiwan”, it should be “3. Division of Clinical Research, Department of Medical Research and Division of Medical Oncology, Center for Immuno-oncology, Department of Oncology, Taipei Veterans General Hospital, Taipei, Taiwan, 4. National Yang Ming Chiao Tung University School of Medicine, Taipei, Taiwan”.

In the published article, an author name was incorrectly written as “Sean M.N. Wong”. The correct spelling is “Natalie S.M. Wong”.

In the published article, there was an error in the Funding statement. An acknowledgement was missed. The correct Funding statement appears below.

## Funding

This work was supported in part by the Chang Gung Memorial Hospital CMRPG3J0971~3 CORPG3J0251~3. We thank to Zai Lab (Taiwan) Limited for the educational grant to Taiwan Society of Molecular Medicine. The funder Zai Lab (Taiwan) was not involved in the study design, collection, analysis, interpretation of data, the writing of this article, or the decision to submit it for publication.

The authors apologize for these errors and state that this does not change the scientific conclusions of the article in any way. The original article has been updated.

## Publisher’s note

All claims expressed in this article are solely those of the authors and do not necessarily represent those of their affiliated organizations, or those of the publisher, the editors and the reviewers. Any product that may be evaluated in this article, or claim that may be made by its manufacturer, is not guaranteed or endorsed by the publisher.

