# Supplementary material for: Compassionate Use of Ripretinib for Patients With Metastatic Gastrointestinal Stromal Tumors: Taiwan and Hong Kong Experience
Source: Front Oncol. 2022 Jun 29;12:883399. doi: 10.3389/fonc.2022.883399 (PMC9277435; doi:10.3389/fonc.2022.883399)
Supplement: Supplementary Table 1 — Univariate analysis of prognostic factors in progression-free survival and overall survival. [file Table_1.docx]

Supplementary table 1. Univariate analysis of prognostic factors in progression-free survival and overall survival

| Variables | Total no. | Progression-free survival | | | |  | Overall survival | | | |  |
| --- | --- | --- | --- | --- | --- | --- | --- | --- | --- | --- | --- |
|  |  | No. of  event (%) | Median  (months) | 95 % CI | *P*  value |  | No. of event (%) | Median  (months) | 95 % CI | *P*  value | |
| Gender |  |  |  |  | 0.033 |  |  |  |  | 0.224 | |
| Male | 16 | 13 (81.2) | 10.7 |  |  |  | 5 (31.2) | NA |  |  | |
| Female | 4 | 1 (25.0) | 5.2 | 3.0–7.4 |  |  | 0 | NA |  |  | |
| Age (years) |  |  |  |  | 0.602 |  |  |  |  | 0.231 | |
| <=60 | 8 | 6 (75.0) | 6.1 | 3.8–8.5 |  |  | 3 (37.5) | NA |  |  | |
| >60 | 12 | 8 (66.7) | 5.7 | 1.4–9.9 |  |  | 2 (16.7) | NA |  |  | |
| ECOG |  |  |  |  | 0.100 |  |  |  |  | 0.129 | |
| 0/1 | 16 | 11 (68.8) | 6.7 | 1.9–11.5 |  |  | 3 (18.7) | NA |  |  | |
| 2 | 4 | 2 (75.0) | 4.0 | 1.3–6.7 |  |  | 2 (50.0) | 3.2 | – |  | |
| BMI |  |  |  |  | 0.818 |  |  |  |  | 0.690 | |
| <18 | 2 | 2 (100.0) | 5.7 | – |  |  | 1 (50.0) | 6.0 | – |  | |
| 18-27 | 15 | 11 (73.3) | 6.7 | 1.5–11.9 |  |  | 3 (20.0) | NA |  |  | |
| >27 | 3 | 1 (33.3) | 1.7 | – |  |  | 1 (33.3) | NA |  |  | |
| Albumin (g/dL) |  |  |  |  | 0.048 |  |  |  |  | 0.022 | |
| <=3.5 | 11 | 9 (81.8) | 4.0 | 0.4–7.6 |  |  | 5 (45.5) | NA |  |  | |
| >3.5 | 9 | 5 (55.6) | 10.7 | 5.9–15.5 |  |  | 0 | NA |  |  | |
| Lymphocyte |  |  |  |  | 0.055 |  |  |  |  | 0.456 | |
| <=2432 | 18 | 12 (66.7) | 6.7 | 3.3–10.1 |  |  | 4 (22.2) | NA |  |  | |
| >2432 | 2 | 2(100.0) | 2.4 | – |  |  | 1 (50.0) | 3.2 | – |  | |
| NLR |  |  |  |  | 0.227 |  |  |  |  | 0.791 | |
| <=1.55 | 3 | 1 (33.3) | NA |  |  |  | 1 (33.3) | NA |  |  | |
| >1.55 | 17 | 13 (66.5) | 5.7 | 3.8-7.5 |  |  | 4 (23.5) | NA |  |  | |
| MLR |  |  |  |  | 0.190 |  |  |  |  | 0.941 | |
| <=0.21 | 4 | 2 (50.0) | 10.7 | 0.1–23.2 |  |  | 1 (25.0) | NA |  |  | |
| >0.21 | 16 | 11 (7.5) | 5.7 | 3.0–8.3 |  |  | 4 (25.0) | NA |  |  | |
| PLR |  |  |  |  | 0.003 |  |  |  |  | 0.074 | |
| <=86.44 | 3 | 3 (100.0) | 2.4 | 2.2–2.6 |  |  | 2 (66.7) | 3.2 | 3.2–3.3 |  | |
| >86.44 | 17 | 11 (64.7) | 6.7 | 2.7–10.7 |  |  | 3 (17.6) | NA |  |  | |
| Location |  |  |  |  | 0.702 |  |  |  |  | 0.267 | |
| Stomach | 8 | 6 (75.0) | 5.7 | 3.9–7.4 |  |  | 1 (12.5) | NA |  |  | |
| Small bowel | 10 | 7 (70.0) | 4.0 | 0.1–8.5 |  |  | 4 (40.0) | NA |  |  | |
| Others | 2 | 1 (50.0) | 6.1 | – |  |  | 0 | NA |  |  | |
| Best response |  |  |  |  | <0.001 |  |  |  |  | 0.330 | |
| PR | 5 | 4 (80.0) | 8.4 | 8.3–8.6 |  |  | 0 | NA |  |  | |
| SD | 7 | 2 (28.6) | NA |  |  |  | 2 (28.6) | NA |  |  | |
| PD | 8 | 8 (100.0) | 2.4 | 1.7–3.2 |  |  | 3 (37.5) | NA |  |  | |
| Any toxicity |  |  |  |  | 0.430 |  |  |  |  | 0.003 | |
| No | 6 | 4 (66.7) | 5.2 | 0.1–11.3 |  |  | 4 (66.7) | 3.2 | 0.1–6.8 |  | |
| Yes | 14 | 10 (71.4) | 6.7 | 3.0–10.4 |  |  | 1 (7.1) | NA |  |  | |
